# Using optical mapping data for the improvement of vertebrate genome assemblies

**DOI:** 10.1186/s13742-015-0052-y

**Published:** 2015-03-18

**Authors:** Kerstin Howe, Jonathan MD Wood

**Affiliations:** Wellcome Trust Sanger Institute, Wellcome Trust Genome Campus Hinxton, Cambridge, UK

**Keywords:** Optical mapping, Genome Reference Consortium, reference genomes, genome assembly, Rmap

## Abstract

Optical mapping is a technology that gathers long-range information on genome sequences similar to ordered restriction digest maps. Because it is not subject to cloning, amplification, hybridisation or sequencing bias, it is ideally suited to the improvement of fragmented genome assemblies that can no longer be improved by classical methods. In addition, its low cost and rapid turnaround make it equally useful during the scaffolding process of *de novo* assembly from high throughput sequencing reads. We describe how optical mapping has been used in practice to produce high quality vertebrate genome assemblies. In particular, we detail the efforts undertaken by the Genome Reference Consortium (GRC), which maintains the reference genomes for human, mouse, zebrafish and chicken, and uses different optical mapping platforms for genome curation.

## Introduction

### Optical Mapping

‘Optical mapping’ is a term originally coined for a method to produce ordered restriction maps by optical inspection and sizing of restriction fragments created from single linearised DNA molecules. It was first described for yeast, and has since been applied to generate maps of bacteria, eukaryotic parasites, plants and vertebrates [1]. The creation of single-molecule restriction maps (Rmaps) is followed by a series of analyses, ultimately resulting in the creation of a genome-wide map. Optical maps can be aligned to an *in silico* digest of a proposed genome sequence, allowing segments of the sequence to either be confirmed or flagged for future attention.

Optical mapping techniques have been applied both in the creation and the refinement of vertebrate genome assemblies. Whilst the initial approaches focussed on quality checking of selected genome regions, gap sizing, placement of previously unlocalised contigs and variation detection, the applications now extend into *de novo* sequence assembly creation and the investigation of methylation profiles [2,3].

### The Genome Reference Consortium

The Genome Reference Consortium’s (GRC) mission is to maintain and improve the reference genomes of human, mouse, zebrafish and chicken by correcting errors, filling gaps and representing variation [4,5]. The GRC uses optical mapping generated on automated platforms for reference genome improvement. Its adoption in genome curation has had a major influence on the human reference assemblies GRCh37 and GRCh38, the mouse reference assemblies GRCm37 and GRCm38 and the zebrafish reference assembly GRCz10. The creation of optical maps within the consortium to support current and future curation is ongoing. At the same time, optical mapping data is used for the *de novo* generation of mouse strain assemblies (Keane T, personal communication) to be included in future GRC efforts.

## Review

### Vertebrate genome assembly assessment with optical mapping data

One of the earliest applications of optical mapping analysis was in the resolution of the *DAZ* locus on human chromosome Y [6], comprising a then unknown number of *DAZ* genes. At that time, genome assemblies were primarily created by selecting and sequencing BAC clones, ordered on a chromosome tiling path, generated by restriction digest mapping. Due to the unordered nature of the individual restriction fragments, the resulting maps were often imperfect and the resolution of complex regions often failed. Visual inspection of the inherently ordered Rmaps, created through optical mapping, enabled ordering and orientation of 16 highly repetitive clones initially identified as belonging to the *DAZ* locus via hybridisation. This revealed four very similar *DAZ* genes, residing in the genome in two pairs in an inverted tandem arrangement. Despite the power of the method, which was further documented through its repeated application to bacterial and plant genomes, and the advances towards its automation [7], its use did not become widespread in the vertebrate community until 2008. At this time, optical mapping was successfully used to confirm eight large insertions identified by fosmid one-end-anchoring to the human reference assembly NCBI35 [8]. This analysis was performed on an automated platform and involved assembling individual Rmaps into consensus maps, which could subsequently be aligned to an *in silico* digest of the reference genome, covering 95% of the reference sequence.

The first vertebrate genome to be comprehensively improved by using automated optical mapping was the mouse MGSCv3 draft assembly, leading to the release of the much improved NCBIm36 reference assembly [9]. The authors reported the remarkable value of the data in placing and ordering assembly components, particularly in highly repetitive and peri-centromeric regions. To achieve this, individual Rmaps were assembled into consensus maps and aligned to the *in silico* digested reference sequence assembly*.* The consensus maps showed 99% similarity to the sequence assembly, but highlighted 423 discordant regions, each of which was manually reviewed. This resulted in 95 assembly corrections, pertaining to re-finishing incorrect component sequences to address deletions and insertions of several kB in length, the removal, addition or exchange of individual components, and the change of component order. The consensus maps also covered two thirds of the remaining gaps; this enabled gap sizing and provided valuable information for future recruitment of sequence data into those regions. Next, the technology was applied to the human genome again to create optical consensus maps for three lymphoblastoid-derived cell lines and a complete hydatidiform mole, in a process termed ‘iterative assembly’ [10]. Here, optical maps were created by alignment of individual Rmaps to an *in silico* digested reference assembly and subsequent iterative reference-free assembling of those and the remaining Rmaps. The resulting optical consensus maps spanned up to 98.6% of the human reference assembly NCBI35 and detected notable structural variation in the individual cell lines. The optical map analysis also helped to identify 322 errors in NCBI35 and sized 183 gaps, verified by comparison with the improved GRCh37 assembly. The study demonstrated a strong concordance between optical mapping and both fosmid end sequencing and paired-end mapping when detecting indels relative to the reference assembly. It also showed how optical mapping can complement other classical methods of genome assembly analysis, such as microarray analyses and tiling array CGH, for instance by revealing the genomic structure of identified large sequence gains.

More recently, optical mapping has been used to resolve discrepancies between two existing cow genome assemblies, UMD3.1 and Btau4.6. This involved the creation of a large-scale optical map by combining initially reference-guided iterative assembly [10] and *de novo* assembly of Rmaps using the software Seed & Mature (SAM, a de Bruijn graph-based assembly approach), the visualisation of discordances between optical map and sequence assembly with the help of the software ‘Genome Polysemy and Synonymy’ (GPS), and subsequent manual curation (Schwartz DC, personal communication).

The increasingly automated solutions used in the studies described above relied on either the production of optical maps in the Schwartz laboratory, or on the commercial OpGen Argus platform [11,12]. An alternative method to create optical mapping data is realised in BioNano Genomics’ Irys platform, which uses microfluidics to draw single DNA molecules through microchannels, past a sensor that detects fluorophores incorporated after treatment with a nicking endonuclease [13,14]. This platform couples high throughput data collection involving multiple labelling and analysis with *de novo* map assembly [15]. The Irys platform has been used to create haplotype-resolved maps of the human major histocompatibility complex (MHC) region for the BAC clones from the PGF and COX libraries [16]. This revealed an error in the GRCh37 COX region, confirmed by sequencing of the implicated clones, and also demonstrated the use of optical mapping in scaffolding *de novo* assemblies of the MHC region, and in detecting structural variation. The Irys platform has also been used to create optical maps of clones from a haploid hydatidiform mole library (CHORI-17) [17]. The information gathered led to a significant improvement of the complex human 1q21.1-q21 region, subsequently represented in the GRCh38 reference assembly, and established the haploid gene number (289) of the *NBPF* gene family.

### Using optical mapping data with whole genome *de novo* sequence assembly

The studies described so far established optical mapping as well suited to improving assemblies created by sequencing individual clones. These were usually ordered with the help of long-range structural data (e.g. fingerprint contig mapping, genetic mapping, hybridisation), which were expensive and time-consuming to generate. With whole-genome sequencing becoming faster and cheaper due to the advent of high-throughput technologies, optical mapping was recognised as a comparably fast and low-cost complement to provide long-range information.

In an approach combining curation of existing assemblies and the contiguation of *de novo* assemblies, the rat reference genome sequence was improved through large-insert mate pair library-assisted re-scaffolding of the RGSC3.4 reference, and optical consensus maps were used to confirm observed discordances [18]. Optical mapping was also used, not for improving but simply validating, the long- and short-range accuracy of the *de novo* genome assemblies produced for a budgerigar, a Lake Malawi cichlid and *Boa constrictor* during the second Assemblathon [19].

The first *de novo* vertebrate genome assembly created purely from short-read sequencing and optical mapping data was that of a domestic goat [2]. OpGen’s Argus platform and Genome-Builder pipeline were used to automatically and iteratively scaffold contigs into the resulting 2.66 GB genome. Notably, this did not involve the assembly of large-scale optical consensus maps, but the iterative alignment of individual Rmaps to the existing sequence contigs. This reportedly shortened the process from months to days. At the time of writing, the chicken genome reference assembly has been improved by re-scaffolding Galgal4.0 with PacBio RS II sequence, and is now further enhanced with the help of optical mapping analysis and subsequent manual curation, using the same platform and mechanism (Graves T, personal communication).

A recent return to de Bruijn graph-based *de novo* assembled optical consensus maps using the software Germinate & Grow [20] is reported for the automated enhancement of two out of three budgerigar Illumina-PacBio hybrid assemblies. The alignment of the consensus maps to *in silico* maps of the sequence assemblies facilitated iterative scaffolding, leading to a modest reduction in scaffold numbers but substantially improved N50 scaffold size [21].

### How the Genome Reference Consortium uses optical mapping data

The GRC actively explores platforms and strategies for the improvement of the reference genomes in its care beyond the simple generation of additional clone sequences. Optical mapping is one such valuable data type that has been used to make substantial changes to the structure of these genomes. The GRC has access to these data for all the current reference genomes, both those provided by collaborators and produced in-house using the OpGen Argus platform. The GRC has been provided with maps produced from three human cell lines (GM10860, GM15510 and GM18994), and the C57BL6J mouse strain based on digestion using the *Swa*I restriction enzyme [9,10]. OpGen has provided a map from the human cell line NA12878 using the *Spe*I restriction enzyme [12]. Furthermore, the GRC at the Wellcome Trust Sanger Institute has produced its own optical mapping data for the C57BL6J mouse strain generated with *Kpn*I, and for the Tübingen zebrafish strain generated with *Bam*HI.

The highly contiguous nature of the reference genome assemblies of both human and mouse has allowed for easy identification of problematic regions with the long-range information provided by optical mapping. This has been used to identify and aid the correction of a range of issues from simple clonal deletions and over-expanded gaps, to complex rearrangements such as those corrected on human chromosomes 6, 9 and 10. Optical mapping has also allowed for detailed placement of BAC clones initially localised to chromosomes through admixture mapping analysis [22,23]. With the human reference genome assembly comprising sequences from multiple clone libraries and whole genome shotgun assemblies of individual DNA sources, the benefit of having multiple maps can be seen by distinguishing true assembly errors from variant loci. In addition, having multiple optical maps generated from different restriction enzymes allows for increased coverage across the genome by providing information missing in one map through an absence of restriction sites. Despite the absence of variation in the mouse reference genome, the additional optical mapping analysis by the Wellcome Trust Sanger Institute complemented the existing data from the Schwartz laboratory in an effort to detect discordance caused by incomplete digest, and to provide a mapping framework in regions lacking target sites for one of the used restriction enzymes.

For zebrafish, the GRC has taken a different approach to using optical mapping data. Whilst still being a traditional clone assembly, the genome remains in a comparably discontiguous state due to its high repeat content and complexity [24]. With ambiguous placement of numerous contigs caused by conflicting or absent meiotic map marker information, a linking approach for the sequence contigs of the whole genome was needed, and for this OpGen’s Genome-Builder pipeline has been used [2]. This pipeline takes the genomic sequence contigs and aligns Rmaps to either end of each sequence contig through an iterative process. It then looks for overlaps in the aligned Rmaps in an attempt to link contigs together with an optical map ‘bridge’. This process of contig joining allows for the accurate placement of ambiguous sequences, and led to the discovery of numerous misassemblies in the form of intra- and inter-chromosomal rearrangements in the reference. In addition, it highlighted problems with the existing order and orientation of anchored sequence contigs. This scaffolding approach employed by Genome-Builder is now being utilised by the GRC to improve short-read *de novo* whole genome assemblies, aided by the long-range mapping information it provides. It is currently being applied to *Mus musculus castaneus* and *M. spretus*.

**Figure 1:**
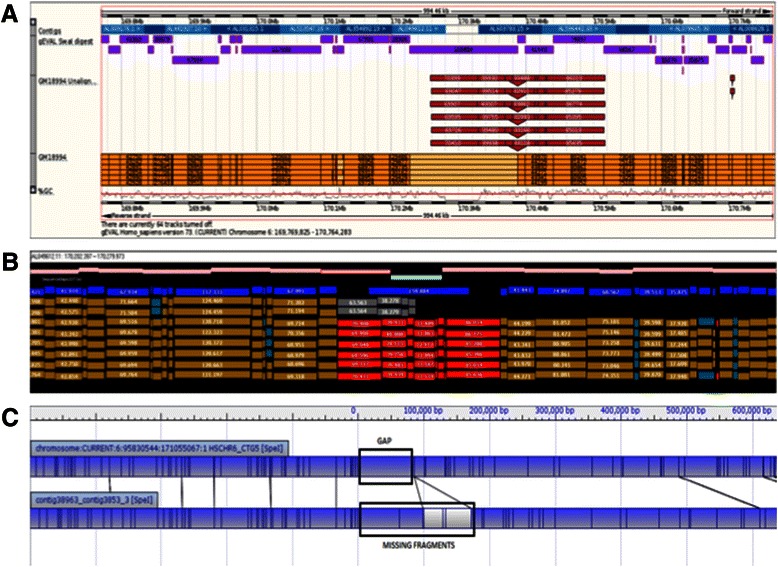
**Comparison of viewers for optical mapping data aligned to a region on chromosome 6 in GRCh37 featuring a sequence gap. (A)** Optical consensus maps of cell line GM18994 (*Swa*I digest) [10] in gEVAL [25]. The yellow track shows the aligned optical map fragments. Red inserts show fragments present in the optical map but absent from the reference. The virtual digest of the reference sequence is added in purple for comparison. **(B)** Gnomspace viewer [10] showing the same region and optical mapping data. Unaligned fragments are depicted in red. **(C)** OpGen’s Mapsolver alignment of an optical consensus map of cell line NA12878 (*Spe*I digest) to the same region. The upper track shows the virtual reference digest with the sequence gap indicated. The lower track shows the optical map including the currently missing fragments.

For viewing optical map alignments, each platform has its own proprietary software (Figure 1). The Schwartz laboratory has developed the GnomSpace viewer [10], while OpGen has developed Mapsolver [12]. Both of these viewers allow for the identification and inspection of genome assembly problems. GnomSpace is a fast, lightweight viewer, which displays optical map alignments against the reference genomes’ clone tile paths, enabling easy interpretation of problematic regions and precise pinpointing of their locations. In contrast, OpGen’s Mapsolver software has no tile path information, but the global alignments it creates for each chromosome facilitate the resolution of complex rearrangements. The ability to import and align new or unlocalised sequences greatly improves the ability to integrate these into the reference assemblies. In addition, the GRC curators are also applying optical mapping visualisation software to improve highly repetitive regions where sequence alignments remain inconclusive and optical mapping data might be absent. Here, *in silico* digests of sequence contigs are produced and the contigs are then ordered with the help of (for example) MapSolver, without the need to perform actual mapping experiments.

**Figure 2:**
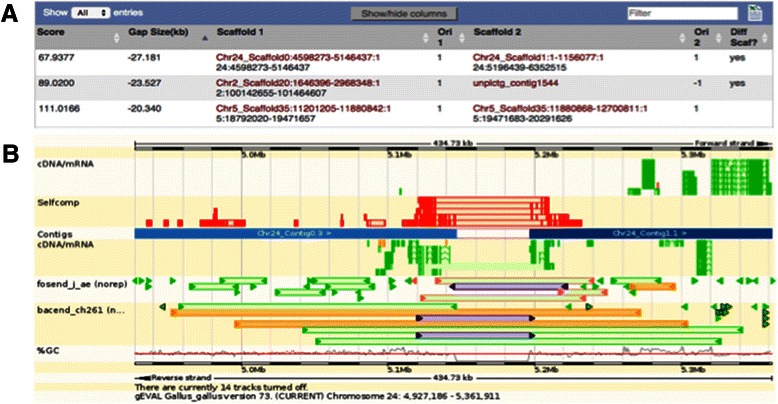
**GenomeBuilder results of optical mapping analysis of the chicken genome assembly Galgal4.1 visualised in gEVAL [**25**]. (A)** List view of possible joins. A negative gap size indicates that the currently separated scaffolds should overlap. **(B)** Genome view of first listed issue showing the current gap and additional evidence to support an overlap of the neighbouring scaffolds. The self comparison of genome sequence, cDNA alignments and BAC/fosmid end alignments indicate repeated sequence around the gap. Repetitive end alignments of the same BAC/fosmid end are highlighted in purple. Incorrect distance between ends of the same BAC/fosmid are highlighted in orange.

The complete optical maps for human and mouse have been imported for display in the Sanger Institute’s Genome Evaluation Browser gEVAL [25]. This integration facilitates the assessment of regions of interest through comparison between each optical map cell line and the wealth of other data the browser offers, such as BAC library end sequence alignments, cDNA alignments and comparison to other assemblies. This gives both GRC genome curators and external users the ability to see all the available evidence in problematic regions of the genome. The gEVAL browser also supports lists of issues that can be run through to support systematic curation (Figure 2).

In addition to the OpGen Argus platform, more recently the GRC has had access to BioNano Genomics’ Irys platform for optical mapping [13]. The GRC is currently working with a map produced by BioNano Genomics, which is aiding improvements in the CHM1*tert* hydatidiform mole platinum reference genome assembly [17,26]. Further optical maps for genomes of interest to the GRC will be produced on this platform.

## Conclusions

Optical mapping provides genomic long-range information free from sequence-specific bias that might influence cloning, DNA amplification or probe-selection for hybridisation, and can be applied to complex regions. It is therefore ideally suited to confirm and complement results gathered by other long-range strategies to generate genome assemblies – namely fingerprint-contig and genetic mapping strategies, as well as fosmid end-sequence placement and mate-pair analyses. As such, it provides data to troubleshoot and resolve genome issues as well as variation information. As optical consensus maps can be generated without a reference, unlike many other approaches, optical mapping detects insertions as easily as deletions, whilst at the same time providing sizing and restriction maps of the missing sequence. It has therefore been used successfully to quality-check and extend/improve existing assemblies, in addition to being integrated into pipelines to produce *de novo* sequence assemblies.

As this review has demonstrated, although optical mapping is 20 years old, the maturing of automated platforms and software that work on a gigabase scale has led to an ever-widening uptake in the field of vertebrate genome sequencing in the last few years. In particular, it is valuable in assembly creation, where an increasing number of software solutions are being developed to integrate optical mapping data into assembly pipelines. The refinement of existing assemblies is a more manual process and therefore limited to those groups able to commit to providing the required resources. One of these groups is the Genome Reference Consortium, which has access to both of the currently available commercial platforms.

Despite the advances made in optical mapping technology, there remain unresolved problems and future opportunities. The mapping data provided are of relatively low resolution; this has the advantage of low data storage costs, but the variability of the detected size for identical fragments combined with possible incomplete enzymatic reactions, means that the creation of a single optical map assembly from individual Rmaps remains an informatics challenge. Consensus maps based on repeated alignments to a reference genome can provide valuable information to confirm or correct a given sequence contig, and often reach far into gaps or even bridge them. However, only a single *de novo* assembly of all Rmaps provides the necessary means to improve complex genomic regions where the sequence is currently sparse. *De novo* optical map assemblies have been produced by the Schwartz laboratory, and also by the commercial providers of optical mapping platforms, but were originally developed for smaller genomes. Consequently, when applied to vertebrate genomes, they encounter scaling issues demanding excessive runtime and memory. To our knowledge, there are also currently no reports of software available for high throughput vertebrate variation detection based on optical mapping data – a clear potential area for future use.

The limited number of publications on the use of optical mapping in vertebrates might be due to limited public awareness of its existence, as these data are still not easily obtainable and useable by the public. Whilst several records of optical mapping analyses for bacteria and plants have been submitted to Genbank, e.g. *Medicago truncatula* MAP_000014.2 [27], no such submissions seem to exist for vertebrates. All current submissions appear to be restricted to the OpGen/Schwartz approach and include an ordered list of restriction fragments and additional information in XML format. It is currently unclear as to which format submissions from other platforms will take. Until recently, the lack of submissions also resulted in a lack of optical mapping presence in genome browsers. This is now remedied by the display of optical mapping information in gEVAL and the availability of a GRC trackhub, enabling the display of an increasing number of optical mapping datasets used for curation.

In summary, optical mapping is a valuable extension to the existing genomic toolkit. Given the growing market of commercial platforms and an anticipated expansion of software solutions, we can expect it to have a bright future in vertebrate genome sequencing and human variation detection for genomic medicine.

## Abbreviations

Rmap: single-molecule restriction map
GRC: Genome Reference Consortium
